# Disentangling direct and indirect effects of water availability, vegetation, and topography on avian diversity

**DOI:** 10.1038/s41598-018-33671-w

**Published:** 2018-10-19

**Authors:** Vladimír Remeš, Lenka Harmáčková

**Affiliations:** Department of Zoology and Laboratory of Ornithology, Faculty of Science, Palacky University, 17. listopadu 50, 77146 Olomouc, Czech Republic

## Abstract

Climate is a major driver of species diversity. However, its effect can be either direct due to species physiological tolerances or indirect, whereby wetter climates facilitate more complex vegetation and consequently higher diversity due to greater resource availability. Yet, studies quantifying both direct and indirect effects of climate on multiple dimensions of diversity are rare. We used extensive data on species distributions, morphological and ecological traits, and vegetation across Australia to quantify both direct (water availability) and indirect (habitat diversity and canopy height) effects of climate on the species richness (SR), phylogenetic diversity (PD), and functional diversity (FD) of 536 species of birds. Path analyses revealed that SR increased with wetter climates through both direct and indirect effects, lending support for the influence of both physiological tolerance and vegetation complexity. However, residual PD and residual FD (adjusted for SR by null models) were poorly predicted by environmental conditions. Thus, the FD and PD of Australian birds mostly evolved in concert with SR, with the possible exception of the higher-than-expected accumulation of avian lineages in wetter and more productive areas in northern and eastern Australia (with high residual PD), permitted probably by older biome age.

## Introduction

**Figure 1:**
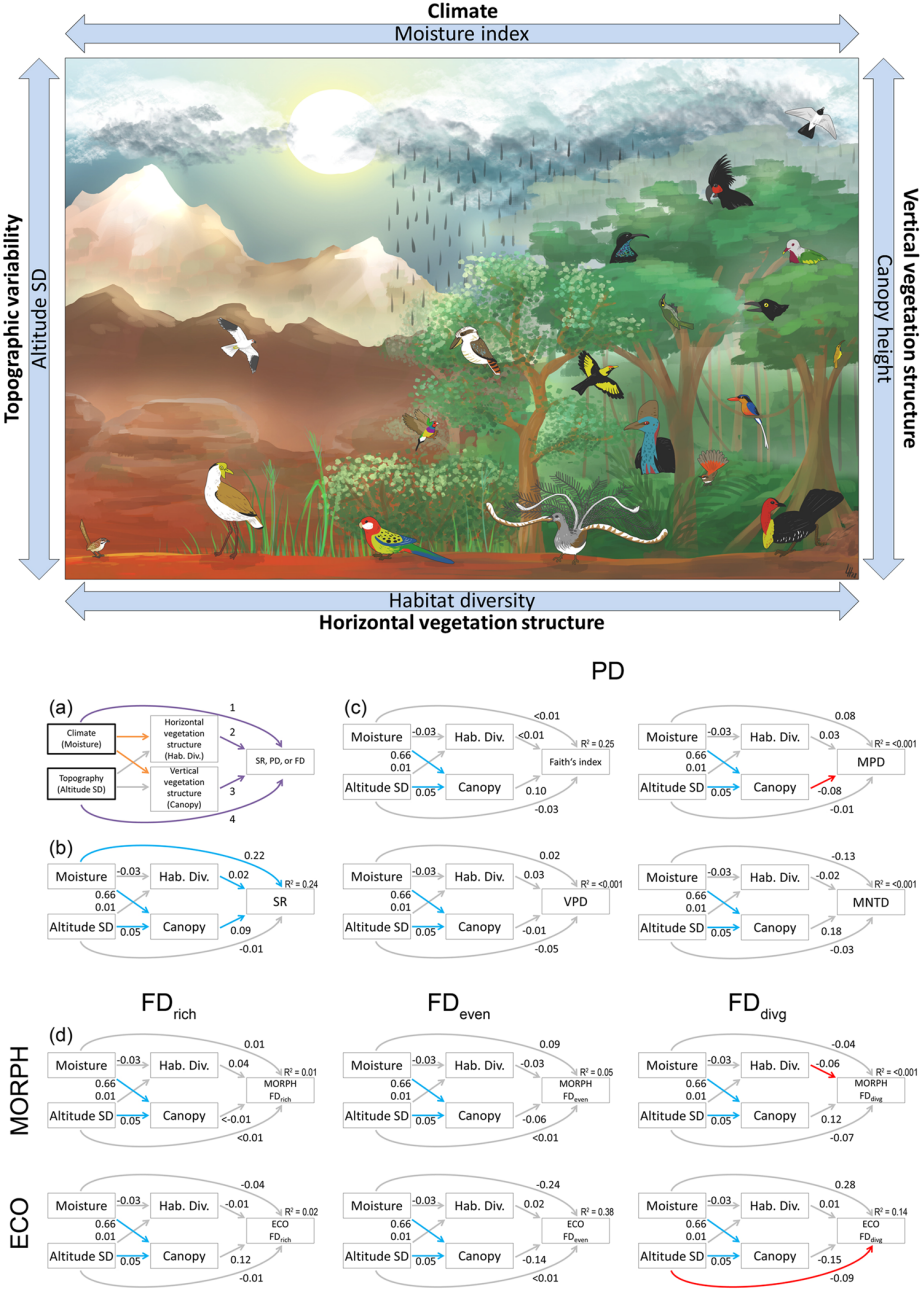
Results of path analyses. Top figure (drawing by L. Harmackova) and panel (a) show the conceptual framework to quantify direct and indirect effects of climate (water availability quantified by moisture index) on species richness (SR), phylogenetic (PD), and functional diversity (FD). In panel (a) climate can affect diversity either directly due to species physiological tolerance (1: the physiological tolerance hypothesis^4^) or indirectly by facilitating richer vegetation, which in turn provides more niches^5,6^. More niches can arise by means of higher horizontal vegetation diversity (2: habitat diversity^11^) and/or higher vertical vegetation diversity (3: canopy height^7,8^). We also include a direct effect of a major source of abiotic heterogeneity (4: topographic heterogeneity defined as the Standard Deviation (SD) of Altitude^13^). Abiotic factors are in thick box. Colour codes are as follows: Magenta = direct effects of variables on diversity; Orange = direct effects of climate on vegetation structure; Grey = other logical links between variables. For details, see the main text. (**b**–**d**) Results of path analyses for SR (**b**), residual PD (**c**), and residual FD (**d**). Blue arrows are statistically significant positive effects, red arrows are significant negative effects, and grey arrows are nonsignificant effects. Numbers along paths are standardized effect sizes for individual paths. Numbers above response variables are pseudo-Rsq values, which were the same for Hab. Div. (<0.01) and Canopy (0.37) in all path models and thus are not depicted in the figure.

Current climatic conditions, especially energy and precipitation, are major determinants of species richness^1–3^. This effect can be direct, due to a subset of species tolerating harsh climates (the physiological tolerance hypothesis)^4^ or indirect due to wetter and warmer environments facilitating more complex habitats providing more ecological niches (the vegetation structure hypothesis)^5,6^. These indirect effects include taller vegetation with increased vertical vegetation complexity^7–9^, more habitat types^10,11^, and possibly greater food availability (e.g. invertebrate biomass)^12^. All these effects suggest that there is more available energy supporting more individuals, which allows more populations to potentially co-exist with densities sufficient to avoid local stochastic extinction. In addition, these effects might be modified by spatial heterogeneity resulting in an increase in microhabitats, which could further influence diversity (e.g. topographic heterogeneity^13^; see Fig. 1a). Both direct and indirect climatic effects on species richness (SR) have been demonstrated on spatial and environmental SR gradients^9,12,14–18^. However, effects of climate on residual phylogenetic diversity (PD) and functional diversity (FD) adjusted for SR have been quantified only rarely^19–21^, and studies comparing both direct and indirect effects of climate on residual PD and FD are even rarer^22^.

A comprehensive assessment of all dimensions of diversity should provide a new insight into the origin and maintenance of diversity^23–28^. For example, climatic tolerance could limit the number of species capable of colonizing a challenging environment (e.g. desert) leading to low SR, but these species could ecologically diversify to fill available niches, which would lead to high residual FD^29^. Moreover, although SR might increase with ecological factors, e.g. productivity, residual FD might be better predicted by the evolutionary time available for species differentiation, as is the case in mammals globally^30^. Additionally, climatic history can complicate inference as climate change can drive diversity. For example, areas with faster climate change during the late Quaternary were taxonomically poorer in amphibians, birds, and mammals^31^. Additionally, regions with long existence and climatic stability were biologically diverse^32,33^ and areas with greater climatic stability since the last interglacial period (last 125 000 years) had higher residual PD in birds globally^28^. Furthermore, the FD of European plants and PD of European dragonflies were lower than expected for given SR in northern areas of the European continent due to the legacy of glacial climate change^34–36^. It is clear that multiple dimensions of diversity should be considered when exploring the processes influencing the distribution of diversity in space.

Australia is uniquely suited to resolving the direct and indirect effects of climate on the distribution of biodiversity. This continent experienced dramatic changes in its environment involving intense aridification in the past 15–20 million years^37–39^. Species-rich and phylogenetically diverse assemblages are usually situated in the remnants of the original wet forests on the east coast^40^ existing from at least the early Paleogene ca. 55 Mya^39^ and probably even from before the separation of Australia from the rest of Gondwana some 80 Mya^41,42^. However, the Australian arid zone (now comprising ca. 70% of the continent) also provided an opportunity for the extraordinary diversification of many lineages of animals and plants, which required multiple adaptations to challenging abiotic conditions^37^. The Australian arid zone was invaded relatively recently, which was demonstrated by phylogeny-based studies confirming the mesic origins of arid-adapted lineages^38,43^. Moreover, FD might also be elevated in certain lineages in the arid zone. For example, local assemblages of reptiles in Australian deserts are much more diverse in terms of both SR and functional type than anywhere else in the world^44^ and certain species even seem to fulfil the ecological roles of insects or mammals^45^. Furthermore, the SR of Australian honeyeaters (Meliphagidae), which originated ca 25 Mya in Australian wet forests^46,47^ decreases towards dry areas^43^ and only five lineages have become endemic for the arid zone^48^. However, their ecological and behavioural (foraging behaviour) diversity does not decline as fast with aridity as their SR, showing that the arid-adapted species have ecologically diverged to a similar degree as their mesic counter-parts^29^.

To disentangle direct and indirect effects of climate on biodiversity in an ancient and rich radiation of vertebrates, we studied patterns in SR, PD, and FD in Australian and Tasmanian birds across major climatic and environmental gradients. We used four PD indices and three FD indices based on three sets of ecologically important traits linked to ecological niches (breeding habitat, diet, and foraging substrate) to build on previous studies^40^ and investigate the effects of water availability and available niche space on SR, PD and FD. Specifically, we (i) mapped the geographical distribution of the residual PD and residual FD of Australian and Tasmanian birds adjusted for SR by null models and identified areas deviating from our null expectations, and (ii) fitted path models to link SR and residual PD and residual FD to water availability, topography, canopy height, and habitat diversity. By using path models (Fig. 1a), we quantified both the direct and indirect effects of a major climatic variable driving Australian environments, namely water availability, to provide comprehensive insights into the processes determining the accumulation and maintenance of biodiversity in Australian and Tasmanian avifauna. It should be noted that we focused only on the current climate and environment as potential explanations for bird diversity, because finding data of sufficient detail and scope for past climates and environments was not possible.

## Materials and Methods

### Data

To map PD and FD in assemblages we created a geographical grid with a 1 × 1 degree resolution (longitude x latitude; equivalent to ca. 10,000 km^2^) across Australia and Tasmania in R software ver. 3.4.3^49^. We eliminated cells in which land constituted less than 50% so that the final grid consisted of 692 cells, where one grid cell represented one assemblage. We used the distribution ranges of mainland Australian and Tasmanian species of birds (n = 536) obtained from^50^ to generate presence-absence data for all species in each assemblage. We used only breeding ranges where the species were extant and the area of the range occupied at least 10% of a cell. The assemblages were then characterized by the list of species present in each cell. For our continent-wide analyses, we preferred coarse-grained range extents rather than point occurrence records, e.g. from The Atlas of Living Australia (www.ala.org.au), because the former are less susceptible to sampling bias that might compromise data on local scales^51^. Phylogenetic trees for the computation of PD indices were obtained from the publicly available archive at birdtree.org (Hackett constraint)^52^. We created one Bayesian maximum clade credibility tree out of 500 phylogenies using TreeAnnotator software embedded in BEAST^53^. However, to ensure that phylogenetic uncertainty did not compromise the calculation of PD indices, we calculated all PD indices (see below) across 100 randomly chosen phylogenetic trees to quantify the variation in indices across trees.

We used species morphology and ecology to quantify FD. As dimensions of the species’ ecological niche we used following three traits: (i) type of breeding habitat, (ii) diet, and (iii) foraging substrate. Data on morphological and ecological traits for every species were obtained from The Handbook of Australian, New Zealand and Antarctic birds^54–60^. Morphology included mean body mass (grams) and the length of wing, tail, tarsus, and bill (mm). Type of habitat was divided into 12 categories (forest, woodland, shrubs, savanna, grassland, reed, swamps, sand, freshwater, marine, rocks, and human settlement), diet into eight categories (plant material, fruit, nectar, seeds, insects and other invertebrates, fish, other vertebrates, and carrion), and foraging substrate into four categories (ground, vegetation, air, and water). Each category of each ecological trait received a value ranging from 0 to 5 according to the information in^54–60^. The value symbolized the proportional use by the species (e.g. 0 – not used, 2.5 – the given category represented one half of the use, 5 – only this category was used) so that the sum of all values in all categories in a given ecological trait for a given species was always equal to 5. It should be noted that using a scale from 0 to 5 was arbitrary and any similar scale would serve equally well (e.g. a percentage scale from 0 to 100).

To test both the direct and indirect effects of climate on diversity, we selected four variables as predictors in our analyses: two environmental variables (habitat diversity and canopy height), one climatic variable (moisture index), and one topographic variable (variability in elevation within geographic cells). We obtained these predictors as follows. First, we obtained data on land cover^61^, canopy height^62^, and water availability and elevation (The Atlas of Living Australia, http://www.ala.org.au, accessed 24 April 2017). The land cover dataset provides the proportional cover constituted by particular habitat types expressed in percentages and we used nine out of twelve metrics from this dataset (we removed habitats that occur only marginally in Australia, i.e. needle leaf trees, snow, and open water). Canopy height is the average height of the highest stratum of vegetation in a geographic cell (in metres). We characterized water availability by moisture index, which is the annual mean of the monthly ratio of precipitation to potential evaporation (pan, free water surface). It is a numerical indicator of the degree of dryness of the climate at a given location, whereby high values indicate relatively wet locations while low values indicate relatively dry locations. The Australian arid zone is usually defined by values less than 0.4^37^. We characterized topographic variability by the standard deviation of the elevation in 1,000,000 subcells within each grid cell (Altitude SD henceforth), where the original resolution of the elevation was 3.6 arc-seconds (equivalent to ca. 0.1 km). The original resolution of other datasets was 30 arc-seconds for land cover (equivalent to ca. 1 km), 1 km for canopy height, and 12 arc-minutes for climate (equivalent to ca. 22 km). We rescaled these datasets to match our grid by taking the mean value of the smaller pixels within our 1 × 1 degree mask (equivalent to the resolution of ca. 100 km). Canopy height and climate data were entered into our analyses unaltered. However, we transformed land cover metrics into an index of habitat diversity by computing Levins’ index on the basis of the number of types of land cover and their relative proportions in each grid cell (for more information see)^10^. The values of this index range from zero, representing uniform grid cells dominated by one habitat type, to one, representing diverse grid cells that contain all habitat types represented equally. This metric thus includes information on both the number of habitat types and their proportions in a given cell and is more informative than, for example, the simple number of habitat types in a cell.

We selected the abovementioned predictors on the basis of previous work and strived to use predictors that are easy to understand and whose potential link to biodiversity is easy to interpret. At the same time, we focused on recent environments, because it was impossible to obtain sufficiently detailed information on past habitats and climates to factor them into our analyses. The first predictor, habitat diversity, relates to the number of habitat types and their relative areas; that is, areas with high habitat diversity (Levins’ index → 1) might provide ecological space for more and diverse species^10,11^. The second predictor, canopy height, is expected to be important in that a higher canopy should provide more microhabitats and resources and thus support higher biodiversity^9^. It provides an index of vertical vegetation richness and productivity. The reasons for this are twofold: (i) The number of vegetation strata logically increases with tree height and we verified this assertion by correlating canopy height with leaf area index (LAI), which is defined as the one-sided green leaf area per unit ground surface area (in m^2^/m^2^; The Atlas of Living Australia, http://www.ala.org.au, accessed 24 April 2017). The correlation was sufficiently high (Log LAI vs. Sqrt Canopy height, r = 0.82), confirming our expectation. (ii) Net primary productivity increases with tree mass^63^ and forest stand biomass^64^, which again increases with maximum tree size^65^. The third predictor, topographic variability, is expected to be important in that high topographic heterogeneity (Altitude SD) might provide more niches for species with variable ecological requirements^13^. The fourth predictor, water availability, was previously reported to correlate positively with species richness in a wide range of plant and animal groups, especially on the southern hemisphere^3,40^, and is considered a major driver of biome dynamics in Australia^37,38,66^. Thus, although other aspects of climate might affect diversity, we used the moisture index, which uniquely summarizes water balance by integrating water inputs from precipitation with water losses due to solar energy. An alternative index of water availability is precipitation deficit, which is the monthly difference between precipitation and potential evaporation (pan, free-water surface; The Atlas of Living Australia, http://www.ala.org.au, accessed 24 April 2017). However, correlation between the two indices of water balance was sufficiently high (Log Moisture index vs. Sqrt Precipitation deficit, r = 0.96) and thus we used only the moisture index. The spatial distribution of our predictors across Australia and Tasmania is apparent from Fig. S1 in Appendix S1.

### Phylogenetic and functional diversity

We used indices to cover phylogenetic richness, divergence, and regularity^67^.

1. For richness, we used Faith’s index^23^, which represents overall PD in an assemblage as the sum of branch lengths connecting all species in that assemblage.
2. For divergence, we used the Mean Nearest Taxon Distance index (MNTD)^68^ and Mean Pairwise Distance (MPD)^68^. While MNTD represents the average phylogenetic distance between closest relatives in an assemblage, MPD represents the average pairwise phylogenetic distance among all species^25^. Thus, MNTD is informative for questions related to terminal branching, whereas MPD is informative for questions related to branching occurring deep within a tree^67^. All the three indices mentioned above showed good performance in extensive simulations^69^.
3. For regularity, we used Variation of Pairwise Distances (VPD)^70^, which represents variance in all pairwise phylogenetic distances. It should be noted that we inverted the sign of VPD so that high values mean higher regularity, whereas low values mean low regularity.

We used the functions ‘pd’, ‘mpd’, and ‘mntd’ from the ‘picante’ package ver. 1.6–2^71^ to compute Faith’s index, MPD, and MNTD; and the function ‘taxondive’ from the ‘vegan’ package ver. 2.4–6^72^ to compute VPD in every grid cell (for more information on these indices see)^25^. Although in all analyses we used PD indices calculated on one Bayesian maximum clade credibility tree, we also recalculated all PD indices on 100 randomly sampled phylogenies to evaluate variation in indices stemming from phylogenetic uncertainty. We thus obtained estimates of all PD indices for all 692 geographic cells across 100 phylogenies. We then submitted these 100 sets of estimates to principal component analysis. The first axis from this principal component analysis accounted for 99.9% of variation in Faith’s index, 99.4% in MPD, 99.2% in MNTD, and 90.4% in VPD, showing that potential effects of phylogenetic uncertainty were likely negligible.

We used appropriate indices to account for all three aspects of FD – namely, functional richness, functional evenness, and functional divergence, as proposed in^73–75^ – while at the same time avoiding the poor-quality tree-based functional space indices as recommended in^76^. We calculated all three indices for the morphological traits and ecological characteristics of species (breeding habitat, food, and foraging substrate; see above). We calculated FD on morphology using scaled values (by subtracting the mean and dividing by one standard deviation) to avoid FD being dominated by overall body size. Thus, our FD indices for morphology are based on relative size proportions that are likely to reflect the ecological functions of species^77^.

1. FD richness represents the overall volume of the functional space that is occupied by an assemblage^74^. FD richness is thus expected to be lower in assemblages with less diverse traits and a small number of species, while assemblages with high variability in functional traits and many species should exhibit higher FD richness. We calculated FD richness as the convex hull volume, which provides an n-dimensional measure of the volume of trait space occupied by species in an assemblage^78^. For ecological characteristics, we used the first five PCoA axes to calculate FD richness^76^.
2. FD evenness measures how regularly the functional space is filled by species^74^. The FD evenness algorithm creates the minimum spanning tree that links the species in the functional space and quantifies their distances from each other on the branches of the tree. Small values of FD evenness represent clustered distances between species while high values represent an even distribution of species on the minimum spanning tree and in the functional space.
3. FD divergence quantifies the dispersion of species in the trait volume^74^. The FD divergence algorithm creates a centre of gravity of all species in an assemblage and calculates their mean distance from this centre. FD divergence is then computed as the sum of deviations of species from the mean distance divided by the absolute value of the same quantity, standardized by the mean distance (see)^74^. High values of FD divergence are thus the result of assemblages with species that are widely dispersed in trait space, and not clustered near the assemblage trait centroid. We used the ‘dbFD’ function from the ‘FD’ package ver. 1.0–12^79^ to calculate FD richness, evenness, and divergence.

### Null models

**Figure 2:**
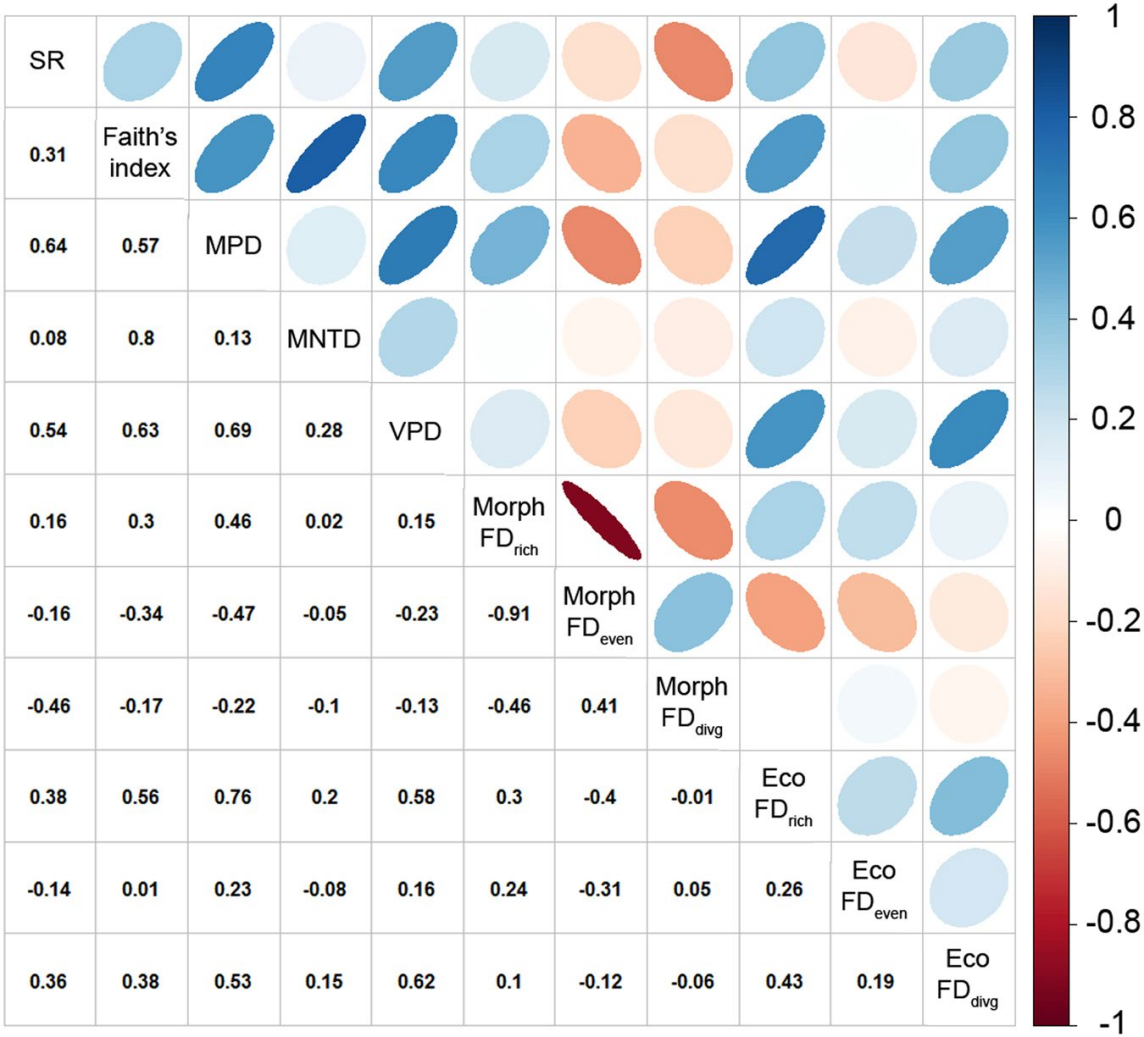
Mutual correlations between species richness (SR) and Standardized Effect Sizes (SES) of phylogenetic and functional diversity (FD) indices (FD_rich_ is functional richness, FD_even_ is functional evenness, and FD_divg_ is functional divergence). Blue colour represents a positive correlation, while red colour denotes a negative correlation. The higher the correlation is, the thinner is the corresponding oval. Numerical values represent Pearson’s correlation coefficient. It should be noted that (i) seemingly missing correlation ovals signal zero correlation (white colour) and (ii) we inverted the sign of SES for the variation of pairwise distances (VPD) so that high values mean higher regularity, whereas low values mean low regularity. Abbreviations not explained above: Morph = morphology, Eco = ecological traits, MPD = mean pairwise distance, and MNTD = mean nearest taxon distance.

Some of the indices correlate with SR by definition and were shown to do so by simulations in previous studies (PD: Faith’s index and MNTD^69^; FD: FD richness^74,75^). Thus, we corrected these indices for SR using null models (see below). Other indices have previously been shown by simulation to be largely independent of SR (PD: MPD^69^; FD: FD evenness and FD divergence^74,75^). However, these indices showed (nonlinear) relationships with SR in our data, or showed correlations between the variance of the index and SR (Fig. S2 in Appendix S1). Thus, to account for these empirically observed correlations with SR and for the sake of applying a consistent approach across all indices, we corrected all indices of PD and FD to ensure they were independent of SR (see also)^80^. We computed Standardized Effect Sizes (SES) for every grid cell (assemblage) as the difference between the observed value and the mean of the expected values divided by the standard deviation (SD) of the expected values. Thus, values of SES higher than 1.96 or lower than −1.96 are outside those expected by chance. To obtain the expected values of each index in each assemblage, we randomly generated samples of species from the pool of all Australian species. In particular, we used the SIM3 model from^81^, whereby species number per site is fixed and all species are equiprobable, which has good overall performance when combined with Faith’s index, MPD, and MNTD^69^. We performed SIM3 by shuffling species names either on the phylogeny for PD indices or in the trait data for FD indices and computed the indices with this randomized phylogeny or trait matrix (while the number of species in an assemblage remained unaltered, see above). We repeated this procedure 1000 times and then computed the mean and SD of the expected values of indices from these simulated data. Mutual correlations between SES of all indices together with species richness are shown in Fig. 2. In addition, to show the sensitivity of results to using null models, we also provide results of analyses where null models were used only in indices where the correlation of mean index value with SR was theoretically expected (Fig. S4 in Appendix S2).

### Statistical analyses

To test both the direct and indirect effects of climate, we used structural equation modelling (SEM) where the SES value of the PD or FD index was the main variable to be explained and four other variables (see above and Fig. 1a) were linked in hypothesized causal relationships in the SEM^82^. To fit the SEM we used a piecewise approach in which the causal relationships were statistically defined and evaluated as mutually interconnected equations^83^. Specifically, we used the following three equations: Habitat diversity ~ Moisture index + Altitude SD; Canopy height ~ Moisture index + Altitude SD; and FD/PD index ~ Moisture index + Altitude SD + Habitat diversity + Canopy height. We used generalized least-squares to account for spatial autocorrelation and we chose the autocorrelation function with the lowest AIC value. We checked the autocorrelation of residuals to ensure that spatial effects were accounted for (Fig. S3 in Appendix S1). We calculated pseudo-Rsq values using the ‘rsquared’ function (the ‘piecewiseSEM’ package ver. 1.2.1)^83^ for R software. All variables were tested for normal distribution; they were log_10_ or square root transformed if necessary and scaled (so that their mean was zero and standard deviation was one) prior to statistical analyses. As such, effect sizes from SEM were mutually comparable for individual paths. For details of our methodological choices see Appendix S3.

## Results

### Spatial patterns

**Figure 3:**
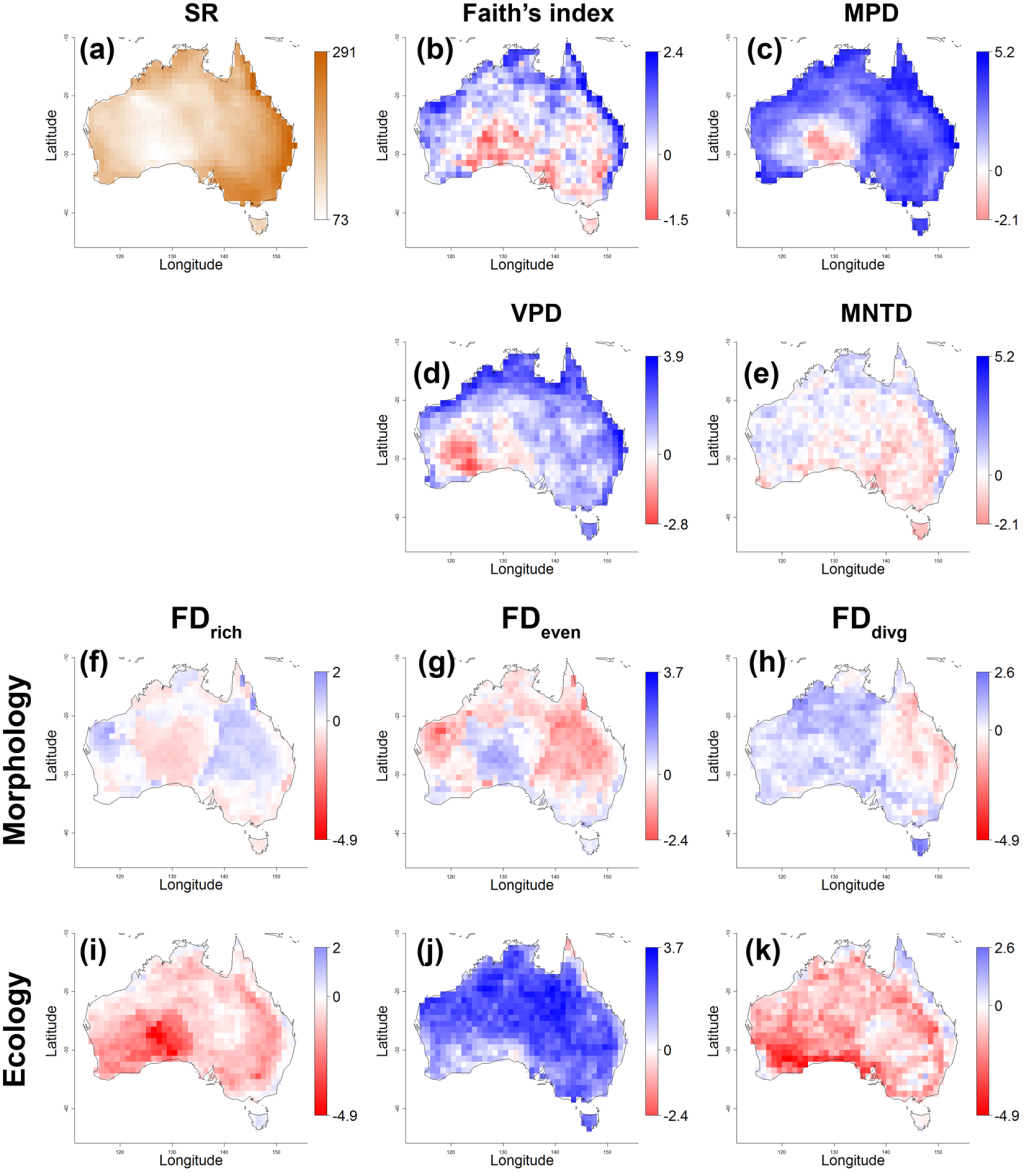
Spatial variation in (**a**) species richness (SR) and Standardized Effect Sizes (SES) of the (**b**–**e**) phylogenetic and (**f**–**k**) functional diversity of Australian birds. Blue colour shows SES values of indices higher than expected by chance for a given species richness, red colour shows values lower than expected. Similar metrics are presented on the same colour scale (mean pairwise distance (MPD) vs mean nearest taxon distance (MNTD), and morphology vs ecology for all functional diversity (FD) indices). It should be noted that (i) values of ca. ±1.96 deviate significantly from null expectations and (ii) we inverted the sign of SES for the variation of pairwise distances (VPD) so that high values mean higher regularity, whereas low values mean low regularity. FD_rich_ is functional richness, FD_even_ is functional evenness, and FD_divg_ is functional divergence.

There was a strong spatial gradient in the species richness (SR) of Australian birds, varying four-fold in 1 × 1 degree squares: it was highest (max. 291 species) in eastern Queensland and New South Wales and lowest (min. 73 species) in west-central deserts (south-eastern Western Australia); it was also low on Tasmania (Fig. 3a). More importantly, when adjusted for SR, the Standardized Effect Sizes (SES) of phylogenetic (PD) and functional diversity (FD) still showed marked spatial gradients, often resembling the gradient in SR (see Fig. 2 for correlations with SR and Fig. 3 for maps of SES). However, these patterns also differed for individual aspects of residual PD and FD, namely richness, divergence, and regularity/evenness. We mention and interpret only SES larger than 1.96 or smaller than −1.96, because only these differ significantly from expected values. Overall residual PD richness (Faith’s index) and residual PD divergence for terminal branches (MNTD) were higher than expected for given SR along northern and eastern coasts (Fig. 3b,e). Residual PD divergence for deeper branches (MPD) and residual PD regularity (VPD) were higher than expected for a given SR again along northern and eastern coasts, while MPD was also higher in central-east Australia (Queensland outback and New South Wales) except for a small area in the southwest interior where MPD and VPD were lower than expected (southern Western Australia; Fig. 3c,d).

The distribution of residual morphological FD was quite irregular across Australia (Fig. 3). The strongest patterns were: (i) a negative correlation between the SES of FD richness and FD evenness (Figs 2 and 3f,g), and (ii) higher than expected residual FD divergence in north-western Australia (mostly Queensland) and on Tasmania (Fig. 3h). The spatial distribution of residual ecological FD richness was marked by exceptionally low values in the arid south-central part of Australia (the Nullarbor Plain and adjacent areas; Fig. 3i). In contrast, residual FD evenness was higher than expected across large parts of Australia (Fig. 3j), while the opposite was true for residual FD divergence across most of southern Australia (Fig. 3k).

### Environmental predictors of SR, residual PD, and residual FD

Path analyses revealed both direct and indirect effects of our environmental predictors on SR (Fig. 1b, Table S1 in Appendix S4). SR increased with increasing water availability (increasing moisture index), higher habitat diversity, and higher canopy. At the same time, canopy height increased with increasing water availability and higher topographic heterogeneity (Fig. 1b, Table S1 in Appendix S4). Taken together, the direct effects of water availability on SR were higher (effect size 0.22) than indirect effects through canopy height (0.06; the magnitude of an indirect effect is the product of the direct effects connecting the two variables, i.e. 0.66 × 0.09), while the opposite was true for topographic heterogeneity, where indirect effects through canopy height, although weak (0.01), were higher than direct effects (zero).

Most aspects of residual PD and FD (i.e., Standardized Effect Sizes) were largely independent of our environmental predictors. The only statistically significant exceptions were (i) decreasing PD divergence for deeper branches (MPD) with higher canopy (effect size −0.08; Fig. 1c), (ii) decreasing morphological FD divergence with increasing habitat diversity (−0.06; Fig. 1d), and (iii) decreasing ecological FD divergence with increasing topographic heterogeneity (−0.09; Fig. 1d). However, some further non-negligible effects approached statistical significance. If we highlight effects with size >0.1 and p-values between 0.05 and 0.07 (Table S1 in Appendix S4), we obtain the following further effects: (i) higher canopy correlates with higher phylogenetic divergence for terminal branches (MNTD, effect size 0.18; Fig. 1c), higher ecological FD richness (0.12), and lower ecological FD evenness (−0.14) and FD divergence (−0.15); and (ii) ecological FD divergence increases with increasing water availability (0.28; Fig. 1d).

## Discussion

Understanding both the direct and indirect effects of climate on phylogenetic (PD) and functional diversity (FD) lags behind our understanding of these effects on species richness (SR). Yet, quantifying these effects is important for a deeper understanding of the origin and maintenance of biodiversity. By studying the spatial distribution of multiple dimensions of the biodiversity of birds across Australia and Tasmania, we demonstrated that avian SR, residual PD, and residual FD all showed strong spatial patterns, most differences being apparent between mesic and arid areas. However, whereas SR correlated with climatic and ecological factors, residual PD and residual FD (adjusted for SR by null models) were mostly independent of these same factors. These results show that both direct (physiological) and indirect (ecological) effects of climate are important in explaining SR at the spatial scale of this study, and reveal important ecological and evolutionary processes determining SR. Furthermore, the residual PD of birds in Australia is much less dependent on these same factors but might be under the influence of historical rather than current factors (e.g. available time), revealing an additional effect of time necessary for the accumulation of lineages in climatically stable and productive regions with old biomes. Lastly, residual FD (morphology, habitats, food, and foraging substrate) behaved spatially rather haphazardly and was not predicted by climate or environment, which suggests that the most relevant ecological and evolutionary effects were already captured by SR.

There were marked spatial gradients in all aspects of the biodiversity of Australian and Tasmanian birds. A conspicuous pattern was high SR in wetter areas along coasts^3,40^, where assemblages were also phylogenetically rich, divergent, and regular (Fig. 3). These findings show that assemblages here are unexpectedly overdispersed in both deeper and terminal branches and that species are unexpectedly evenly spaced in the phylogenetic tree space. This finding is consistent with the higher than expected residual PD in tropical northern and north-western Australia (monsoonal Western Australia, Northern Territory, and Queensland) found in parrots^84^ and birds on the global scale^28^. However, there seems to be limited consistency across classes. Studies variably report patterns of PD similar (Australian mammals)^85^, different (arid-zone lizards and mice in Australia)^21^, and even opposite (mammals globally)^86^ to what we found in birds, making generalizations across taxa difficult. Further complexity was added by spatially inconsistent patterns of residual FD. The only generalization seemed to be the presence of low residual ecological FD in terms of overall richness and divergence in arid southwestern interior areas, which were also typified by very low SR and low residual PD. Overall, the only consistent patterns across all dimensions of diversity seemed to be high SR, residual PD, and some aspects of residual FD in coastal and mesic areas as compared to west-central parts of the arid zone. This seems to be in agreement with the notion of environmental harshness directly limiting the number of lineages that adapt and diversify in challenging conditions^37,43,48^ or with the idea of vegetation structure indirectly driving the number of niches and the amount of energy available^5,6^. We tested both these hypotheses explicitly using path analyses.

When studying the direct and indirect effects of climate on diversity, we included precipitation, which has been demonstrated to be a major driver of biome and vegetation dynamics^66^; SR^40,87^; and clade diversification in Australia (reviewed in^37,38^). Accordingly, we observed positive direct effects of water availability on SR (Fig. 1b), in agreement with previous studies^3^. This effect might have been mediated by the physiological tolerance of species^4^, whereby relatively few species/clades were probably able to invade challenging arid areas, which requires multiple adaptations concerning temperature regulation and water economy^88–90^. Accordingly, evidence indicates that physiological tolerance can at least partly determine species distributions^91–93^. On the other hand, direct effects of water availability on SR are more likely for plants and ectothermic animals, while endotherms such as birds are metabolically more resilient and thus likely to be affected only by rather extreme climatic events^94^. Consequently, an alternative factor might have been the shorter amount of time available for diversification, as the arid zone is comparatively young (15–20 My)^39^; in contrast, more humid, forested habitats were available for a comparatively long time in Australia (at least 55 My, probably even 80 My)^41,42^, and niche conservatism^95^ might have slowed-down the invasion of the arid zone^96^. A role for available time in driving patterns of diversity in Australian birds is supported by our observation of higher residual PD in areas of high SR (Fig. 3), suggesting the accumulation and retention of lineages in climatically stable and highly productive areas that were colonized early^97^. However, these historical effects can be robustly estimated only by using high-quality, dated molecular phylogenies to estimate the timing of the invasion of the arid zone by clades and to map their climatic niche on phylogenies^37,38^, which is yet to be done for the majority of Australian birds^43^, and indeed other clades.

We also identified an indirect effect of climate on diversity – specifically, species richness increasing with the increasing complexity of both horizontal (habitat diversity) and vertical (canopy height) vegetation structure, probably due to complex habitats providing more ecological niches and resources. These results confirm previous studies showing increasing SR with increasing habitat diversity^10,11^. We also confirmed the classical expectation that SR increases with increasingly rich vertical vegetation structure^7^, of which canopy height is a good index (see Methods). This relationship was repeatedly observed on local scales^8,98–100^, although the methodologies of some of these studies have been challenged^101^. These studies were recently extended to continental and global scales, using canopy height as a surrogate of vertical vegetation complexity. So far, results are mixed, with the SR of primates^9^ and amphibians increasing with canopy height^102^, which is not true in birds and mammals^102^. However, the relationships of SR to canopy height varied across continents^102^, suggesting that either the effects of vertical vegetation complexity differ regionally, or that canopy height and vertical complexity correlate only on limited spatial scales – for example, within floristically similar regions. These alternatives remain to be tested.

The east coast of Australia (eastern Queensland, New South Wales, and Victoria) is covered with forests and woodlands and benefits from relatively high rainfall, productivity, and resource availability compared to the arid zone^37,38^. All these conditions are conducive to high SR, confirmed by our path analyses, whereby moist climates both directly and indirectly facilitated high SR in birds. Approximately the same regions were also typified by phylogenetically diverse assemblages. However, perhaps surprisingly, residual PD was not predicted well by habitats or water availability, and we suspect that historical factors may be more important in explaining residual PD. The reason is that forests have been available in eastern Australia since at least the early Paleogene ca. 55 Mya^39^, while the arid zone is comparatively younger, arising in the last 15–20 My^37–39^. Accordingly, lineages in climatically stable environments had time to accumulate diversity and retain old lineages^32,103,104^. Conversely, the drying of the environment might have raised extinction rates, purging SR and PD^105^. Additionally, substantial immigration, origination, and the maintenance of new avian lineages in the new arid habitats could have been inhibited by low productivity and thus low energy availability^106^ or priority effects^107^. However, a limited number of lineages have succeeded even in this challenging environment^37,88^.

These ecological and evolutionary processes are exemplified for instance by Australian honeyeaters (Meliphagidae) and lizards. Honeyeaters originated in wet forests ca 25 Mya^46,47^, enabling the accumulation of SR, and only five lineages subsequently became endemic for new arid environments, this leading to low PD^43,48^. However, these lineages display disproportionately high FD that at least partly offsets the decline in diversity towards arid areas^29^. Similarly, reptiles including lizards are more diverse in moist coastal areas^87^, where many lineages accumulated and diversified; in contrast only a few taxa of arid-adapted lizards (with low PD) diversified and thrived in the new arid zone^108–110^. At the same time, these arid-adapted lizards are exceptionally functionally diverse, which at least partially compensates their limited lineage diversity, and this high functional diversity might have been enabled by a historically contingent lack of functionally equivalent competitors from other animal groups^44,45^. We observed similar effects on the scale of whole Australian avifauna, with the exception of higher residual FD in arid areas.

In summary, in this study we tested both direct and indirect effects of climate (water availability) on bird diversity in Australia and Tasmania. Both direct and indirect effects were significant for SR, as it was well predicted by water availability, habitat diversity, and canopy height. However, residual PD was not related to either climate or habitat; instead, it was probably related to the age of the biome, although this hypothesis remains to be rigorously tested. Analyses of residual FD did not add any further insights beyond the effects captured by SR.

Future studies should find at least six lines of research fruitful. First, improved dated molecular phylogenies will help to robustly estimate historical effects on phylogenetic diversity and the timing of the invasion and accumulation of lineages in the arid zone^37^. Second, simulations should be useful in identifying equilibrium (niches, energy) vs. non-equilibrium (time, diversification rate) drivers of diversity across the aridity gradient in Australia^30^. Third, the investigation of other climatic features, e.g. temperature or seasonality, could shed additional light on their role in shaping diversity. Fourth, although the effects of topographic heterogeneity in our study were negligible, more detailed investigation of altitude, topographic heterogeneity, and related climatic gradients is warranted. Fifth, given the several relationships of residual ecological FD to canopy height and water availability that approached statistical significance in this study, detailed investigation of functional diversity, preferably using more detailed traits and local communities, should prove useful in identifying further ecological and evolutionary processes driving diversity in Australia. Sixth, explicitly evolutionary analyses modelling trait evolution should reveal the potential interplay between species coexistence and trait evolution, especially on local scales where effects of species interactions are expected to be strong.

## Electronic supplementary material

Supplementary information

Dataset

## Data Availability

All primary data sufficient to replicate this study are included in this published article (and its Supplementary Information files).
