# Supplementary material for: Disentangling direct and indirect effects of water availability, vegetation, and topography on avian diversity
Source: Sci Rep. 2018 Oct 19;8:15475. doi: 10.1038/s41598-018-33671-w (PMC6195560; doi:10.1038/s41598-018-33671-w)
Supplement: Supplementary file 1 — Supplementary information [file 41598_2018_33671_MOESM1_ESM.pdf]

Supplementary information for:

Disentangling direct and indirect effects of water availability, vegetation, and topography on avian diversity

Vladimír Remeš* & Lenka Harmáčková*

Department of Zoology and Laboratory of Ornithology, Faculty of Science, Palacky University, 17. listopadu 50, 77146 Olomouc, Czech Republic

***Correspondence**:

Vladimír Remeš or Lenka Harmáčková

Email: vladimir.remes@upol.cz, harmlen@seznam.cz

Contents

Appendix S1. Supplementary figures.

Appendix S2. Sensitivity of results to null models.

Appendix S3. Notes on methodological choices.

Appendix S4. Statistical results of path analyses.

Appendix S1. Supplementary figures.

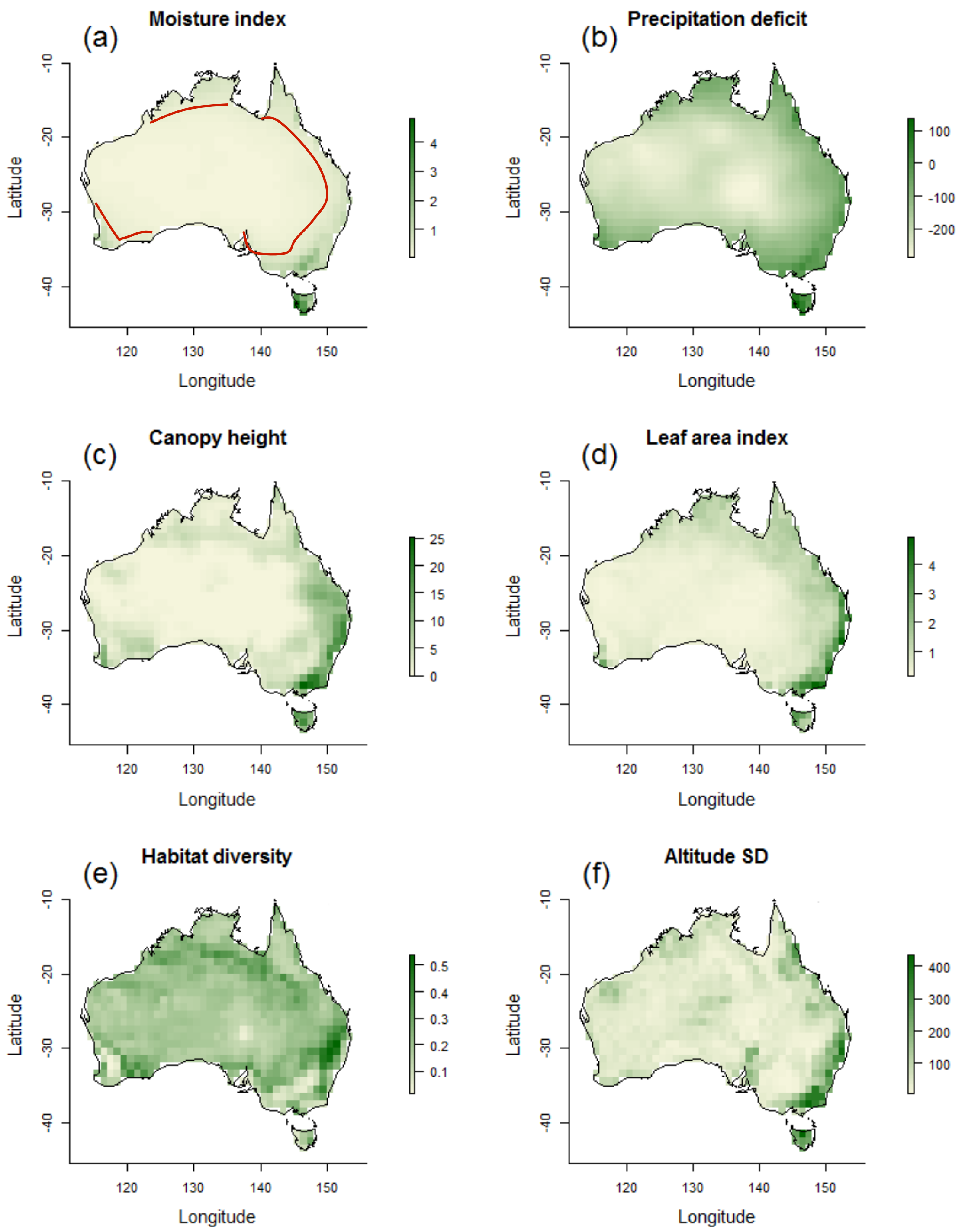

Figure S1. Spatial variation in climatic and environmental variables: Moisture index (a), Precipitation deficit (b), Canopy height (c), Leaf area index (d), Habitat diversity (Levins' index) (e), and Altitude Standard Deviation (f). Red line in (a) represents boundary between arid (values <0.4) and mesic areas.

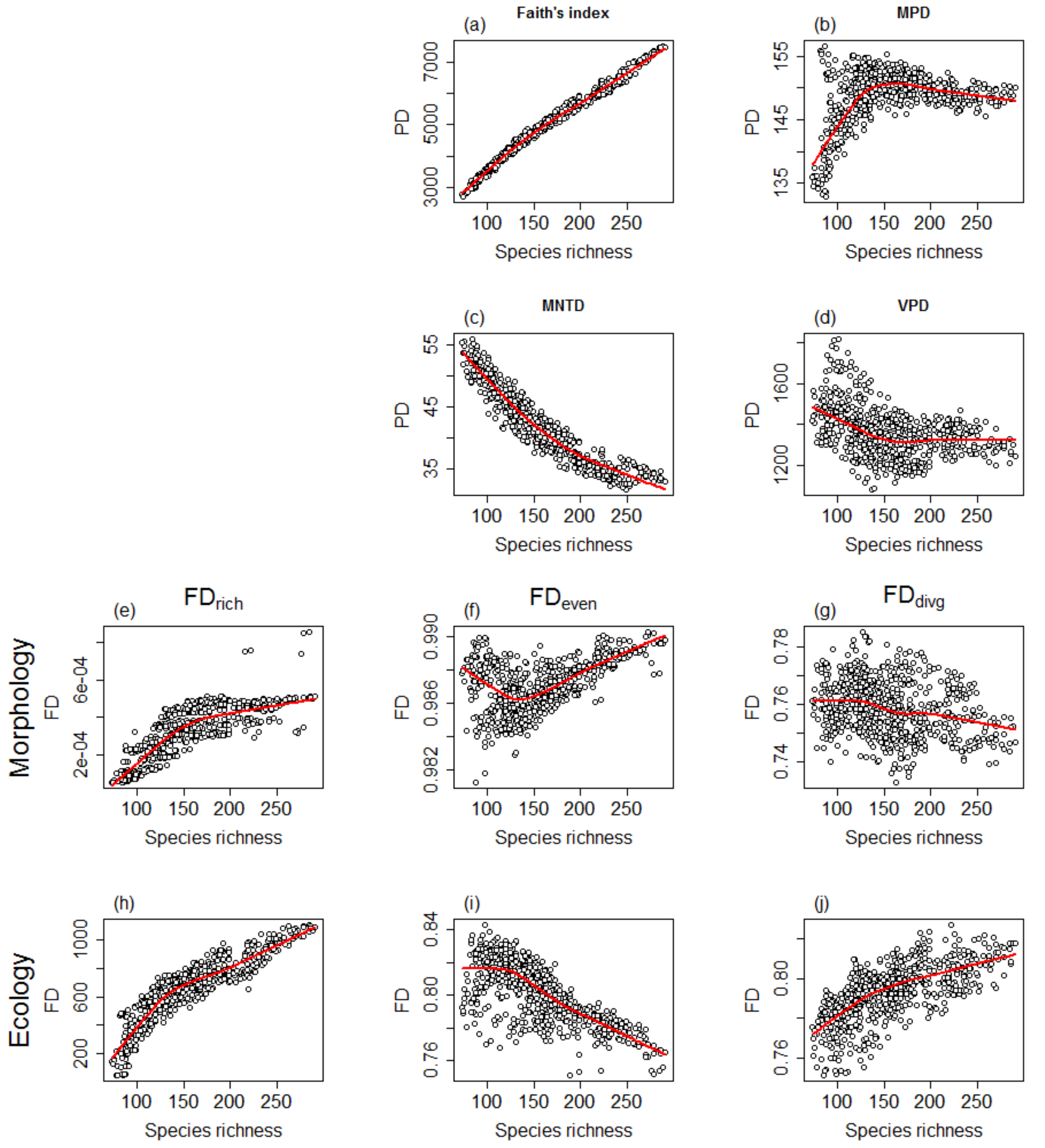

Figure S2. Correlations between species richness and raw phylogenetic and functional diversity indices. These indices were **not** adjusted for species richness by using null models. First two rows show correlations with phylogenetic indices: Faith's index (a), MPD (b), MNTD (c), and VPD (d). Remaining rows represent individual sets of traits: Morphology (e-g) and Ecology (h-j); while columns show functional indices: richness (FD_{rich}; e, h), evenness (FD_{even}; f, i), and divergence (FD_{divg}; g, j).

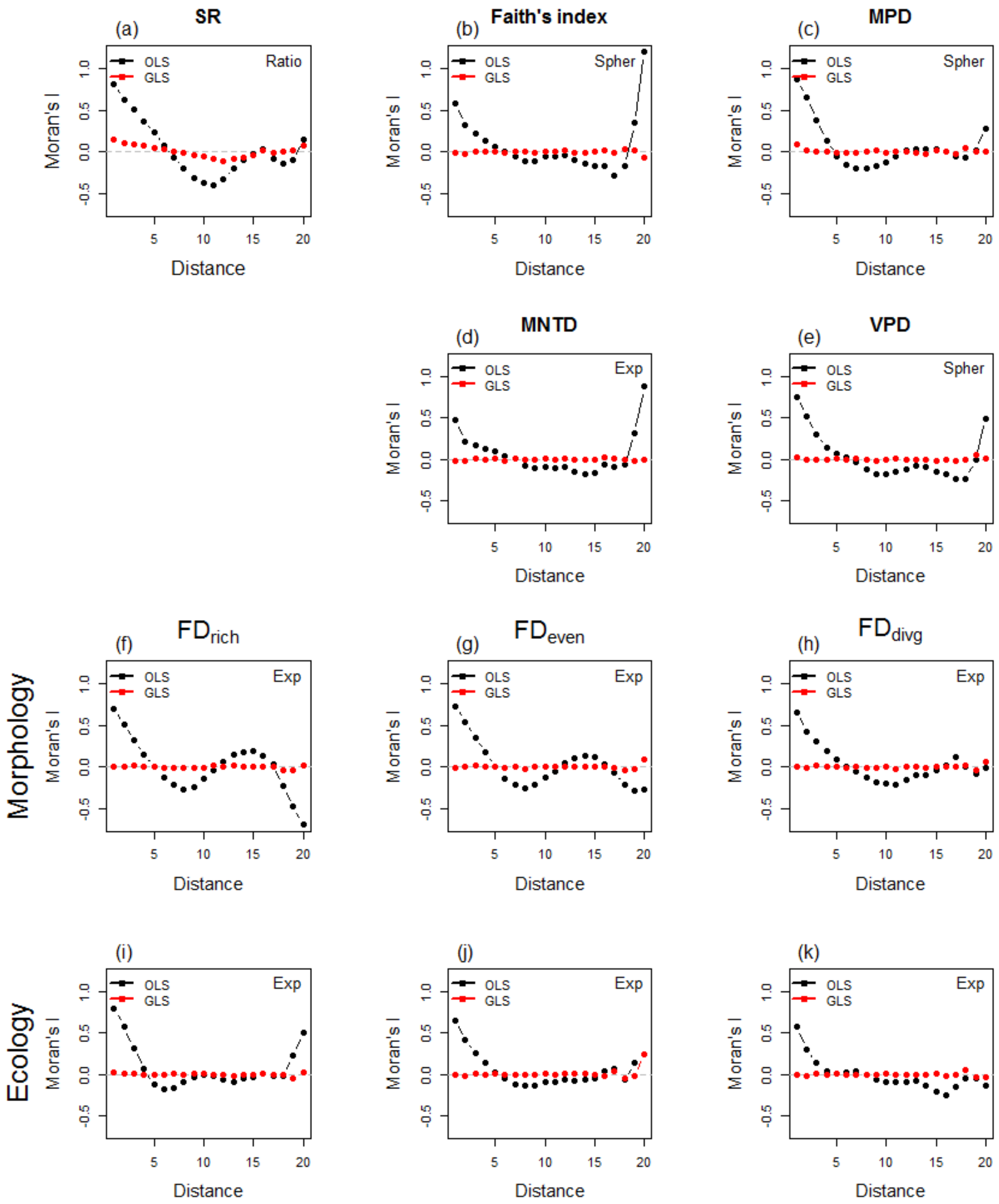

Figure S3. Spatial autocorrelation of residuals of ordinary least-squares (OLS; black) and generalized least-squares (GLS; red) regressions. As an exemplary model we show *SR/PD/FD index ~ Aridity + Altitude SD + Hab. Div. + Canopy height*. For more details on the variables see the main text. Residuals of the SR model are shown in (a), PD models in (b-e), and FD models in (f-k). Abbreviations in top right corner of each panel indicate the spatial correlation structure used in the particular GLS model: exponential (Exp), rational quadratic (Ratio), and spherical (Spher).

Appendix S2. Sensitivity of results to null models

Here we use null models adjusting for SR only for indices that correlate with species richness by definition and were shown to do so by simulations (PD: Faith's index and MNTD [70]; FD: FD richness [75,76]). Other indices were shown by simulation to be largely independent of species richness (PD: MPD [70]; FD: FD evenness and FD divergence [75,76]) or are expected not to be correlated (VPD [71]), and for these indices we use raw values unadjusted for SR in the following analyses. However, please note that this concerns only correlations of mean values of indices, not their variances, because the variance of some indices correlates with SR independent of mean index value (e.g. MPD where variance typically decreases with SR, see Fig. 6.2. in [25] and our Fig. S2b), and thus some bias in analyses might remain even in these latter indices.

To summarize, following indices were adjusted for SR using null models: Faith'sindex, MNTD, and FD richness, whereas following indices were not: MPD, VPD, FD evenness and FD divergence.

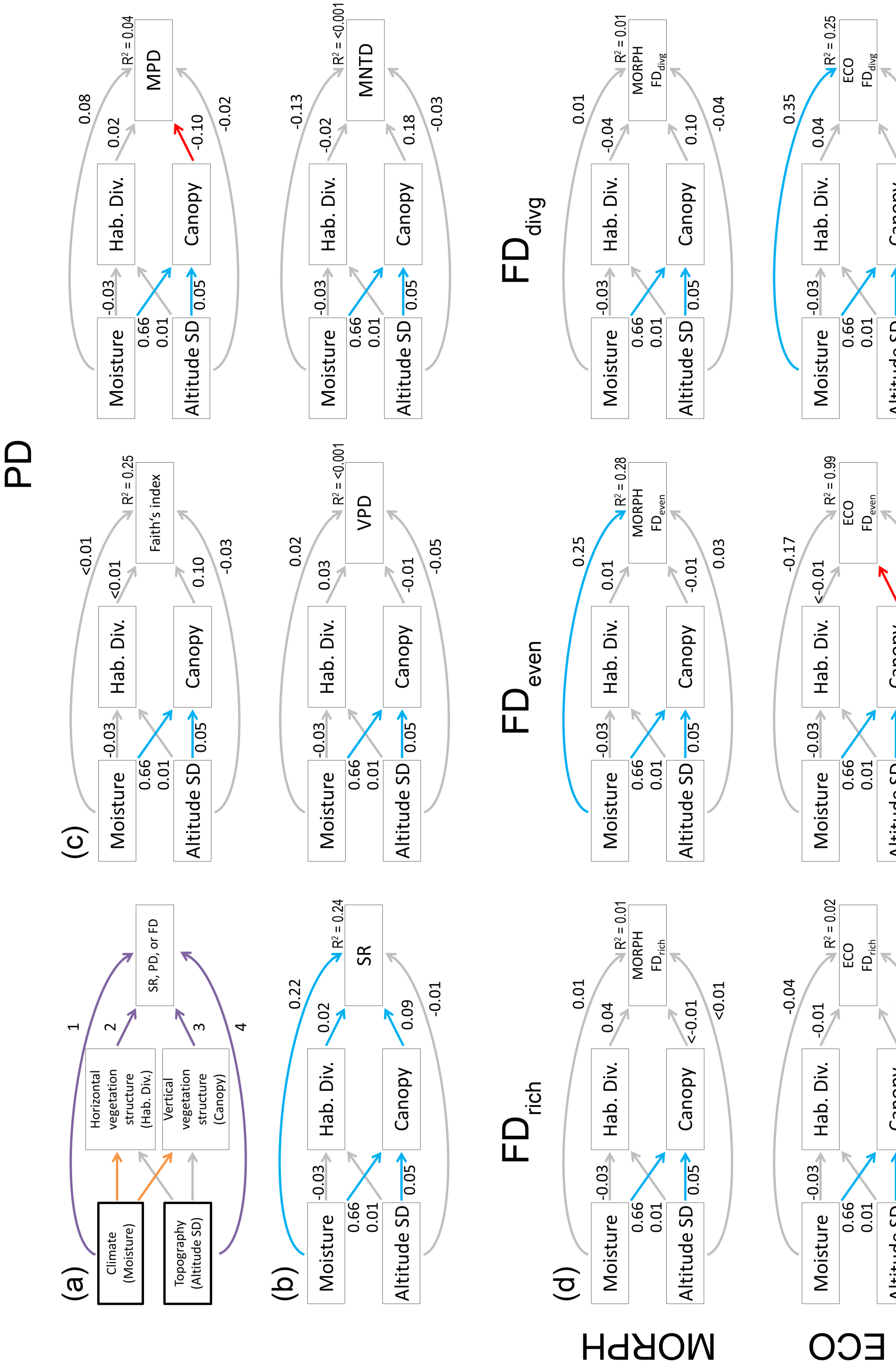

PD
FDrich
FDeven
FDdivg
MORPH
ECO
(a)
(b)
(c)
(d)
Climate (Moisture)
Topography (Altitude SD)
Horizontal vegetation structure (Hab. Div.)
Vertical vegetation structure (Canopy)
SR, PD, or FD
Moisture
Altitude SD
Hab. Div.
Canopy
SR
Faith's index
VPD
MPD
MNTD
MORPH FDrich
ECO FDrich
MORPH FDeven
ECO FDeven
MORPH FDdivg
ECO FDdivg

Figure S4. Results of path analyses. (a) Conceptual framework to quantify direct and indirect effects of climate (moisture index) on species richness (SR), phylogenetic (PD) and functional diversity (FD). Climate can affect diversity either directly due to species physiological tolerance (1: physiological tolerance hypothesis [4]) or indirectly by facilitating richer vegetation, which in turn provides more niches [5,6]. More niches can be brought about by higher horizontal vegetation diversity (2: habitat diversity [11]) and/or higher vertical vegetation diversity (3: canopy height [7,8]). We also include a direct effect of a major source of abiotic heterogeneity (4: topographic heterogeneity defined as SD of Altitude [13]). Abiotic factors are in bold frame. Colour codes are as follows: Magenta = direct effects of variables od diversity; Orange = direct effects of climate on vegetation structure; Grey = other logical links between variables. For details see main text. (b-d) Results of path analyses for SR (b), PD (c), and FD (d). Blue arrows are statistically significant positive effects, red arrows are significant negative effects, and grey arrows are nonsignificant effects. Numbers along paths are standardized effect sizes for individual paths. Numbers above response variables are pseudo-Rsq values, which were the same for Hab. Div. (<0.01) and Canopy (0.37) in all path models and thus are not depicted in the figure.

Appendix S3. Notes on methodological choices.

In our analyses we considered all bird species with current breeding range in Australia. However, that involves also species introduced into Australia quite recently, such as Eurasian blackbird (*Turdus merula*), Eurasian skylark (*Alauda arvensis*) or Scaly-breasted Munia (*Lonchura punctulata*). Previous studies of the effects of introduced species on PD and FD estimates provide mixed evidence. For example, some authors found that PD was indeed sensitive to non-native species, but those were distantly related to original mammal biota [112]. The presence of such species caused great increases in PD. On the other hand, other authors reported that introduced species of plants had actually negative effects on PD [113]. The effect of alien species on calculation of PD and FD indices might thus differ between regions and taxa. We believe that inclusion of recently introduced species did not bias our results from two reasons. First, Australian avifauna consists of over five hundred species; the lowest number of species in an assemblage in our study was 73 and the highest 291. These are considerable numbers compared to twelve introduced species used in our study, especially considering that median number of recently introduced species in one assemblage was three species. Second, most of recently introduced species have close relatives among native Australian birds (e.g. thrushes, larks, estrildid finches) and therefore it is questionable whether their presence in an assemblage should considerably affect calculations of PD and FD values.

References:

112. Davies, T.J. & Buckley, L.B. (2011) Phylogenetic diversity as a window into the evolutionary and biogeographic histories of present-day richness gradients for mammals. *Philosophical Transactions of The Royal Society B*, **366**, 2414-2425.

113. Winter, M., Schweiger, O., Klotz, S., Nentwig, W., Andriopoulos, P., Arianoutsou, M., Basnou, C., Delipetrou, P., Didžiulis, V., Hejda, M., Hulme, P.E., Lambdon, P.W., Pergl, J., Pyšek, P., Roy, D.B. & Kühn, I. (2009) Plant extinctions and introductions lead to phylogenetic and taxonomic homogenization of the European flora. *Proceedings of the National Academy of Sciences USA,* **106**, 21721-21725.

Appendix S4.

Table S1. Statistical results of path analyses. Standardized effects of predictor variables on Habitat diversity, Canopy height, SR, PD, and FD indices. Pseudo-R2 represents marginal coefficients of determination. Since the results of analyses with Habitat diversity and Canopy height as response variables are the same in each path analysis, we show them only once.

	Response	Predictor	Estimate ± SE	P	Pseudo-R^2
	Hab. Div.	log Moisture	-0.03 ± 0.11	0.80	<0.001
		log Altitude SD	0.01 ± 0.05	0.84	
	sqrt Canopy	log Moisture	0.66 ± 0.06	<0.001	0.37
		log Altitude SD	0.05 ± 0.01	<0.001	
SR	log SR	log Moisture	0.22 ± 0.04	<0.001	0.24
		log Altitude SD	-0.01 ± 0.01	0.30	
		Hab. Div.	0.02 ± 0.01	<0.001	
		sqrt Canopy	0.09 ± 0.03	<0.001	
PD	Faith's index	log Moisture	<0.01 ± 0.17	0.98	0.25
		log Altitude SD	-0.03 ± 0.04	0.41	
		Hab. Div.	<0.01 ± 0.03	0.99	
		sqrt Canopy	0.10 ± 0.08	0.24	
	MPD	log Moisture	0.08 ± 0.09	0.36	<0.001
		log Altitude SD	-0.01 ± 0.02	0.64	
		Hab. Div.	0.03 ± 0.02	0.06	
		sqrt Canopy	-0.08 ± 0.04	0.05	
	MNTD	log Moisture	-0.13 ± 0.16	0.43	<0.001
		log Altitude SD	-0.03 ± 0.05	0.55	
		Hab. Div.	-0.02 ± 0.04	0.58	
		sqrt Canopy	0.18 ± 0.10	0.06	
	VPD	log Moisture	0.02 ± 0.11	0.88	<0.001
		log Altitude SD	-0.05 ± 0.02	0.05	
		Hab. Div.	0.03 ± 0.02	0.09	
		sqrt Canopy	-0.01 ± 0.05	0.87	

		Response	Predictor	Estimate ± SE	P	Pseudo-R^2
FD	Morphology	FD_{rich}	log Moisture	0.01 ± 0.13	0.96	0.01
			log Altitude SD	<0.01 ± 0.04	0.96	
			Hab. Div.	0.04 ± 0.03	0.20	
			sqrt Canopy	<-0.01 ± 0.08	0.98	
		FD_{even}	log Moisture	0.09 ± 0.12	0.47	0.05
			log Altitude SD	<0.01 ± 0.04	0.80	
			Hab. Div.	-0.03 ± 0.03	0.26	
			sqrt Canopy	-0.06 ± 0.08	0.45	
		FD_{divg}	log Moisture	-0.04 ± 0.13	0.77	<0.001
			log Altitude SD	-0.07 ± 0.04	0.06	
			Hab. Div.	-0.06 ± 0.03	0.05	
			sqrt Canopy	0.12 ± 0.08	0.11	
	Ecology	FD_{rich}	log Moisture	-0.04 ± 0.12	0.75	0.02
			log Altitude SD	-0.01 ± 0.03	0.79	
			Hab. Div.	-0.01 ± 0.02	0.60	
			sqrt Canopy	0.12 ± 0.06	0.09	
		FD_{even}	log Moisture	-0.23 ± 0.14	0.10	0.38
			log Altitude SD	<0.01 ± 0.03	0.91	
			Hab. Div.	0.02 ± 0.03	0.41	
			sqrt Canopy	-0.14 ± 0.07	0.06	
		FD_{divg}	log Moisture	0.28 ± 0.15	0.06	0.14
			log Altitude SD	-0.09 ± 0.04	0.03	
			Hab. Div.	0.01 ± 0.03	0.82	
			sqrt Canopy	-0.15 ± 0.09	0.08	
